# The Role of GLP1-RAs in Direct Modulation of Lipid Metabolism in Hepatic Tissue as Determined Using In Vitro Models of NAFLD

**DOI:** 10.3390/cimb45060288

**Published:** 2023-05-24

**Authors:** Ana Petrovic, Dunja Igrec, Karla Rozac, Kristina Bojanic, Lucija Kuna, Tea Omanovic Kolaric, Vjera Mihaljevic, Renata Sikora, Robert Smolic, Marija Glasnovic, George Y. Wu, Martina Smolic

**Affiliations:** 1 Faculty of Dental Medicine and Health Osijek, Josip Juraj Strossmayer University of Osijek, 31000 Osijek, Croatia; 2 Faculty of Medicine Osijek, Josip Juraj Strossmayer University of Osijek, 31000 Osijek, Croatia; 3 Health Center Osijek-Baranja County, 31000 Osijek, Croatia; 4 Department of Medicine, Division of Gastrenterology/Hepatology, University of Connecticut Health Center, Farmington, CT 06030, USA

**Keywords:** GLP-1, GLP-1RA, semaglutide, liraglutide, NAFLD, NASH, NASF, in vitro, cell culture, lipid metabolism

## Abstract

Glucagon-like peptide 1 receptor agonists (GLP-1RAs) have been shown to improve glucose and lipid homeostasis, promote weight loss, and reduce cardiovascular risk factors. They are a promising therapeutic option for non-alcoholic fatty liver disease (NAFLD), the most common liver disease, associated with T2DM, obesity, and metabolic syndrome. GLP-1RAs have been approved for the treatment of T2DM and obesity, but not for NAFLD. Most recent clinical trials have suggested the importance of early pharmacologic intervention with GLP-1RAs in alleviating and limiting NAFLD, as well as highlighting the relative scarcity of in vitro studies on semaglutide, indicating the need for further research. However, extra-hepatic factors contribute to the GLP-1RA results of in vivo studies. Cell culture models of NAFLD can be helpful in eliminating extrahepatic effects on the alleviation of hepatic steatosis, modulation of lipid metabolism pathways, reduction of inflammation, and prevention of the progression of NAFLD to severe hepatic conditions. In this review article, we discuss the role of GLP-1 and GLP-1RA in the treatment of NAFLD using human hepatocyte models.

## 1. Introduction

NAFLD is the most common liver disease and globally recognized public health problem, with a rapidly increasing incidence and prevalence [1,2,3]. It is usually associated with T2DM, obesity, and metabolic syndrome; however, the pathogenesis of NAFLD is highly complex and currently explained by the “multiple hit” hypothesis. In summary, the “multi-hit” theory suggests that NAFLD is caused by a combination of genetic and epigenetic, environmental, nutritional, and lifestyle factors that lead to the accumulation of fat in the liver. Pathophysiological processes include the accumulation of lipids in hepatocytes, insulin resistance, dysregulated uptake and synthesis of fatty acids (FA), their oxidation and secretion from the liver, increased hepatic glucose production, and lipogenesis. Further “hits” are considered to be oxidative stress-induced mitochondrial dysfunction, lipotoxicity-induced apoptosis, and inflammation, eventually resulting in progression to non-alcoholic steatohepatitis (NASH), fibrosis (NASF), cirrhosis, as well as hepatic malignancies, and liver failure [4].

During the early years of the 20th century, studies on fat accumulation revealed that certain intestinal factors play a significant role in glucose metabolism and homeostasis by stimulating postprandial pancreatic secretion. These factors were termed “incretins”. The incretin hormone glucagon-like peptide 1 (GLP-1) was discovered, and its insulinotropic activity was described [5,6]. This insulinotropic effect of the gut–endocrine–pancreas axis interrelated pathways is commonly referred to as the “incretin effect”, elucidating the phenomenon of higher secretion of insulin after oral glucose intake compared to intravenously administered glucose in healthy individuals with similar levels of glycemia [7,8]. While intact in patients with normal oral glucose tolerance, the incretin effect is impaired in individuals with glucose and lipid dysmetabolism, such as patients with type 1 and type 2 diabetes mellitus (T1DM and T2DM), obesity, and other related metabolic disorders, including non-alcoholic fatty liver disease (NAFLD) [9,10,11,12,13,14]. Following these findings, a growing number of studies examining the incretins and incretin-based therapy has been published in recent years, providing evidence that the “incretin effect” includes processes beyond merely the entero-insular axis and insulinotropic effects.

GLP-1 is an intestinal peptide hormone, a post-translational product of proglucagon, primarily produced and secreted from the intestinal enteroendocrine L-cells [5,15,16]. GLP-1 receptors (GLP-1R) have been found in α-cells of the human pancreas, several brain regions (responsible for appetite, satiety, and food intake-energy balance), heart and vascular tissue, and the kidneys, lungs, and gastrointestinal tract, as summarized by Nauck et al. [17]. However, the expression of GLP-1R in hepatic tissue remains controversial. In 2006, an immunoblot analysis using an animal model found GLP-1R in isolated murine hepatocytes. Pretreatment with a GLP-1R antagonist, exendin fragment 9–39, abolished the positive effects of GLP-1 in the liver [18]. Recently, Yokomori et al. provided immunohistochemistry (IHC) evidence of GLP-1R in hepatocytes in human liver biopsies. However, due to the conflicting results of similar studies [19], possibly because of technical differences [20], there is an ongoing debate on the presence of GLP-1R on hepatocytes. Evidence of GLP-1R on hepatocytes suggested direct effects of a ligand–receptor interaction; however, the findings are controversial or inconclusive. Effects of GLP-1 in the liver are believed to be mediated by indirect pleiotropic mechanisms rather than the direct stimulation of GLP-1R in the liver [21,22,23,24,25,26]. Despite the controversy surrounding GLP-1 receptor expression in hepatic tissue, GLP-1 receptor agonists (GLP-1RA), have been shown to affect dysregulated pathways of metabolic disorders and, therefore, have become a promising therapeutic option.

Over the last decade, GLP-1RAs, such as exenatide, dulaglutide, liraglutide, semaglutide, and others, have been explored as pharmacotherapy for T2DM and obesity, as well for NAFLD [27,28,29,30,31,32,33]. The beneficial pharmacological effects of GLP1/GLP-1RAs include increasing insulin secretion, suppressing glucagon release, slowing gastric emptying, and enhancing satiety, resulting in an improvement in glucose and lipid metabolism, as well as weight loss, reduced cardiovascular risk factors, and the improvement of non-alcoholic fatty liver disease NAFLD [34,35,36,37,38,39].

While GLP-1RAs have been approved for treatment of T2DM and obesity, they have not been officially approved for treatment of NAFLD. In addition, despite the significant amount of research focused on the role of GLP-1 receptors in glucose metabolism and insulin secretion, there is a relative scarcity of comprehensive reviews synthesizing the effects of GLP-1RAs specifically on hepatic lipid metabolism in NAFLD. A significant number of studies have investigated this topic [32,40,41,42,43]. However, the aim of this article is to provide a more comprehensive review of recently obtained knowledge regarding specifically underlying molecular and cellular mechanisms of GLP1 effects on hepatic lipid metabolism and its potential in the treatment of NAFLD. Moreover, as described in the previous section, it is uncertain whether the decrease in steatosis seen in animal and human studies after treatment with GLP-1RAs is a result of the direct activation of hepatic GLP-1R or an indirect effect. Due to this issue, we have opted out of reviewing most recent in vitro studies to eliminate the pleiotropic effects contributing to GLP-1 results in in vivo studies. To eliminate the extra-hepatic factors, we will provide insight into the recently elucidated molecular and cellular mechanisms of GLP-1RAs in cell culture models of NAFLD, providing evidence of their direct role in the modulation of hepatic lipid metabolism, as well as providing other potential therapeutic targets. We will also summarize the most recently published clinical trials for a comparison and possible explanations of these results, as well as some guidelines that could be translated from in vitro models to clinical trials.

## 2. Role of GLP1 in The Modulation of Lipid Metabolism

Evidence supports a role for GLP-1 as a regulator of lipid and lipoprotein metabolism, even the possibility that GLP-1 may activate two distinct receptors, with one of them being similar or equivalent to the pancreatic receptor and the second one responsible for its lipolytic effects [44,45]. 

Lipid metabolism includes processes, such as lipogenesis and lipolysis, lipid peroxidation (when impaired resulting in lipotoxicity), fatty acid oxidation, cholesterol synthesis, and lipid absorption, suggesting that GLP-1 affects all of these processes which, when dysregulated, contribute greatly to the development or progression of NAFLD [43]. In this section, we review the molecular mechanisms of GLP1-RA effects in the modulation of lipid metabolism in hepatic tissue, predominantly in cell cultures using primary human hepatocytes or continuous cell lines, such as HepG2 or Huh7.

### 2.1. Molecular Mechanisms of GLP1-RA in Modulating Lipid Metabolism in Hepatic Tissue 

Research performed over the past decade has suggested a direct role for GLP1 in hepatic lipid metabolism implying the presence of GLP1R in hepatocytes. In this section, we will review the most recently obtained knowledge regarding the molecular mechanisms that mediate the effect of GLP-1Ras on the modulation of hepatic lipid metabolism by directly regulating aberrant processes, such as lipogenesis and lipolysis, FA β-oxidation, and cholesterol synthesis and secretion, as well as its effects on lipotoxicity.

Pathological lipid accumulation in hepatocytes, hepatic steatosis due to dysregulated glucose and lipid homeostasis, is the hallmark of NAFLD and has been shown to be greatly ameliorated in response to GLP-1RA treatment in numerous animal and clinical studies [32,46]. Earlier studies in humans, animal models, and human hepatocyte cell cultures mainly attributed the GLP-RA-mediated reduction in hepatocyte steatosis to the inhibition of hepatic de novo lipogenesis (DNL) [44,47,48]. Gupta et al. conducted a study on primary human hepatocytes demonstrating that GLP-1RA exendin-4 reduces steatosis and improved mitochondrial function and the survival of hepatocytes. Specifically, this group of researchers provided evidence that GLP1-RAs reduced apoptosis and fatty acid induced-endoplasmic reticulum (ER) stress and induced autophagy of free fatty acids (FFAs) in treated cells. Cell apoptosis is believed to be one of the main mechanisms of NAFL progression to NASH and NASF, suggesting the possibility of GLP1-RA-based treatment of NAFLD. This is also a first report providing evidence that GLP-1R is present on human hepatocytes [21]. Recently, a similar study has been performed with liraglutide using a Huh7 cell culture model of NAFLD confirming the results of the above-mentioned study. Omanovic Kolaric et al. also demonstrated that these hepatoprotective and antisteatotic effects were mediated by the downregulation of lipogenic *PPARγ, ACSL1*, and *SREBP-1c* genes in both NAS and drug-induced steatosis (DIS). These genes are known to be involved in key pathways of lipogenesis and FA metabolism in the liver [26]. Further elucidation of these mechanisms was provided in a recent study [49] with exendin-4 in a HepG2 cell culture model of steatosis. Besides the reduction of lipid content in exendin-4-treated steatotic cells, the presence of exendin-4 also decreased *CPT1A, ACC, DGAT1, SCD1, ApoB, FABP1*, and *FOXA1* gene expression as determined via qRT-PCR. These genes are directly involved in metabolic pathways for hepatic FAs and triacylglycerol (TAG) synthesis, the uptake of circulating FFAs, hepatic FA β-oxidation, and hepatic secretion of VLDL, all of which play a role in the pathophysiology of NAFLD and imply the direct effects of GLP1 on lipid metabolism [50,51]. Perhaps the most significant observation of this study, at least regarding the previously mentioned controversial issue of GLP-1R expression on hepatocytes, were the GLP-1R silencing results. While GLP-1R silencing had no effect on gene expression in the oleic acid induced-steatosis cells, the effect of exendin-4 on gene expression with scrambled siRNA was reversed by GLP-1R silencing, implying that the GLP-1R presence and direct agonist–receptor interaction is required for GLP1RA antisteatotic effects in hepatic tissue. This study concluded that the direct activation of GLP-1R by exendin-4 reduces steatosis in an in vitro model via stimulation of the Wnt/β-catenin signaling pathway and reduces *FOXA1* expression and *FABP1* expression, resulting in decreased FFA uptake [49]. An earlier study found that FOXA1 reduced steatosis in primary cultured hepatocytes and HepG2, and the expression of *FOXA1* was downregulated in both murine models of hepatic steatosis and in human steatotic liver tissue [52,53]. The downregulation of expression of *FABP1* was also reported in a similar study on cell cultures and mouse and human NAFLD tissue samples [53]. However, this could have been an adaptive mechanism against FA uptake and fat accumulation. Another group of researchers conducted a study with a synthetic peptide, AWRK6, a GLP-1RA, using both a murine model and HepG2 fatty liver cell models and an insulin-induced HepG2 insulin-resistant cell culture model. The results showed ameliorated steatosis in HepG2 fatty liver model treated with AWRK6, as well as in vivo. The AMP-activated protein kinase (AMPK) signaling pathway has been shown to be a key mechanism in the regulation of hepatic lipid metabolism and inhibition of lipogenesis in the liver via the downregulation of the expression of lipogenic genes, including acetyl-CoA carboxylase (ACC). [54]. Western blot results suggested the involvement of AMPK/ACC signaling in suppressing de novo lipogenesis and enhancing FA oxidation. Considering the established role of PI3K/AKT pathway [55] in insulin-regulated metabolism and the development of insulin resistance, the phosphorylation levels of these proteins was detected via Western blotting both in vivo and in vitro. The results in both AWRK6 treated models of insulin-resistance showed phosphorylated elevation of PI3K and AKT. To summarize, AWRK6 ameliorated steatosis and regulated lipid and glucose homeostasis, possibly by affecting the PI3K/Akt/AMPK/ACC signaling pathway [56]. A recent study with liraglutide demonstrated the involvement of the SHP1/AMPK signaling pathway in both in vivo and in vitro models of NAFLD [57]. SHP1, also called PTPN6, is a protein that is expressed in epithelial cells, skeletal muscles, and hepatic tissue. It has been recognized as an important modulator of glucose metabolism in the liver and insulin resistance and a negative regulator in the pathogenesis of NAFLD. It has been found to promote lipid accumulation, inflammation, and oxidative stress [58,59]. In both in vivo and in vitro models of NAFLD, SHP1 was found to be significantly upregulated, while AMPK was downregulated. Treatment with liraglutide ameliorated lipid accumulation and showed hepatoprotective effects in vivo and in vitro, providing compelling evidence that these effects of liraglutide were mediated by inhibiting hepatic SHP1, resulting in the activation of AMPK. However, a recent study found that SHP1 had anti-inflammatory effects on NASH [60]. Another study also demonstrated partially conflicting results regarding its role in alleviating hepatic steatosis, possibly mediated via the upregulation of lipogenic PPARγ. However, it was also shown that SHP1 deficiency significantly improved obesity-associated NAFLD in liver-specific SHP1-knockout (KO) mice [61] which is consistent with a previous liraglutide study [57]. The conflicting results from in vivo studies show the need for further investigation of SHP1 and its diverse effects. The PI3K signaling-mediated inhibition of fat mass and obesity-associated (*FTO*) gene expression has also been reported to be directly involved in the protective and antisteatotic effects of GLP-1 in vivo and in vitro in a similar study with exenatide [62].

Important processes involved in hepatic lipid metabolism and cholesterol homeostasis are often impaired in NAFLD and other metabolic disorders. Reverse cholesterol transport (RCT) is a pathway by which cholesterol is transported from extrahepatic cells to the liver for elimination as bile salts through intestines [63]. GLP-1 was demonstrated to beneficially affect this process in vivo. However, the scarceness of in vitro studies on GLP-1RA in the regulation of RCT and cholesterol secretion in NAFLD limits the understanding of molecular mechanisms involved in this process. A recent study investigated the ABCA1 and MAPK/ERK1/2 pathway in the regulation of RCT in mice, as well as in Hepg2 cells treated with high concentrations of glucose. MAPK/ERK1/2 signaling has been implicated in the regulation of many physiological processes, including glucose and lipid metabolism, and its dysregulation has been linked to the development of various metabolic disorders [64,65], while ATP-binding membrane cassette transport protein A1 (ABCA1) is a key protein involved in translocating cholesterol into the extracellular department and hepatic cholesterol transportation [65]. In summary, the presented study demonstrated that liraglutide promote RCT and reduces lipid accumulation in hepatic tissue in vivo, while also suggesting that these effects could be mediated by activating the MAPK/ERK1/2 signaling pathway, resulting in increased ABCA1 expression in HepG2 cells under high-glucose conditions that often lead to NAFLD development and progression [66]. A similar in vitro study with exendin-4 supported the involvement of ABCA1 upregulation in GLP-1RA effects on hepatic cholesterol homeostasis and suggested that CaMKK/CaMKIV/PREB signaling pathway activation is necessary for hepatic ABCA1 upregulation induced by exendin-4 and its reduction in cholesterol accumulation in the hepatocytes. Furthermore, blocking GLP-1R with exendin9–39 cancelled all exendin-4 effects on the upregulation of hepatic ABCA1 and the reduction of cholesterol accumulation, suggesting that the expression of GLP-1R on HepG2 cells is essential for GLP-1RA effects on hepatocytes [20]. The main site of the degradation or catabolism of lipid droplets in the hepatocytes is lysosomes. Cholesterol is either excreted as bile acids or processed into lipoproteins (such as VLDL). Autophagy is a complex pathway by which cytoplasmic content is incorporated into the lysosome for degradation and has been shown to regulate lipid metabolism through the breakdown of lipid droplets, as well as the regulation of intracellular energy homeostasis mediated by the degradation of aberrant organelles and proteins [67]. Liraglutide has been found to decrease hepatic steatosis in vivo and in vitro by enhancing autophagy and lipid degradation by the TFEB-mediated autophagy–lysosomal pathway. Transcription factor EB (TFEB) is an important regulator of lysosome and autophagy pathways [68]. The upregulation of autophagy, demonstrated by enhanced expression of its markers Beclin1, Atg7, and LC3, has been found in response to liraglutide in both murine and cell culture models of NAFLD [69]. In a study specifically regarding the involvement of autophagy in hepatic steatosis in vitro, it was found that GLP-1RAs ameliorated FFA-induced lipotoxic liver cell damage and promoted autophagy resulting in the reduced degeneration of hepatocytes in NAFLD [70]. Due to the role of lysosomal–mitochondrial axis in lipotoxicity [71], the autophagy–lysosomal pathway could explain the GLP1-RA-mediated reduction in oxidative stress and lipotoxicity in NAFLD.

The lipotoxicity of excessive lipid accumulation is one of the hallmarks of the progression of NAFL into NASH, NASF, and liver malignancies. This progression is a complex process, which includes Kupffer and hepatic stellate cell activation in response to the production of oxidative stress-induced inflammatory mediators. Lipotoxicity has been extensively researched and recognized as one of the hallmarks in the pathophysiology of NAFLD/NASH/NASF [72]. Both endogenous GLP-1 and liraglutide have demonstrated protective effects against lipotoxicity in pancreatic islets [73]. However, the role of GLP1-RA in reducing hepatic lipotoxicity and the inflammatory response has recently gained attention [48,74].

Several pathways have recently been identified in mediating the. GLP-1RA beneficial effects on lipotoxicity and inflammation in vitro. In a HepG2 cell culture model of NASH, exenatide ameliorated NASH via the inhibition of pyroptosis, which was demonstrated by reduced levels of its mediator molecules, such as nucleotide-binding oligomerization domain-like receptor protein 3 (NLRP3), caspase-1, and IL-1β, resulting in the significant improvement of NASH [75]. One study on a mouse-isolated primary Kupffer cell (KC) cell culture model of NAFLD treated with liraglutide demonstrated that liraglutide attenuated the mitochondrial dysfunction and suppressed NLRP3 inflammasome activation in KCs, resulting in a significant reduction of IL-1β and TNF-α expression levels [76]. Liraglutide treatment promoted the expression of IL-10 and decreased the expression of IL-12 and TNF-α, as well as modulated Kupffer cells to M2-like activation via the cAMP-PKA-STAT3 signaling pathway in a mouse-isolated primary Kupffer cell (KC) model of NAFLD. Kupffer cell activation and their polarization into an M2 phenotype have demonstrated anti-inflammatory properties required for the development of NASH [77]. Liraglutide also decreased apoptosis and levels of reactive oxygen species (ROS) resulting in the suppressed activation of hepatic stellate cells, which is essential for the progression of NAFL/NASH to NASF [78]. Therefore, these studies significantly contribute to the understanding and acknowledging of GLP1-RAs anti-inflammatory effects. A recent study on a HepG2 cell culture model of NAFLD treated with liraglutide found reduced activation of the NLRP3 inflammasome, as well as mTORC1 signaling inhibition, in response to GLP1-RA treatment [79]. The mechanistic target of rapamycin (mTOR) signaling pathways is involved in the regulation of various cellular processes, such as autophagy, apoptosis, lipid metabolism, insulin resistance, and oxidative stress. Due to its ability to induce autophagy and apoptosis, as well as exhibit anti-inflammatory and anti-proliferative effects, it has shown potential in the treatment of NAFLD-associated HCC (non-alcoholic fatty liver disease-associated hepatocellular carcinoma) and other cancers, further elucidating the anti-lipotoxic and anti-inflammatory properties of GLP-1RAs [80,81].

All of the evidence presented in this section provides a valuable summary of the positive effects of GLP1-RAs on cell culture models of NAFLD, imply their direct hepatic lipid metabolism-modulating properties and synthesizes the most recently elucidated molecular pathways mediating these effects, as well as pointing out other potential as a therapeutic option for the treatment of NAFLD, while also providing recently obtained evidence suggesting the presence of GLP-1Rs on hepatocytes.

### 2.2. GLP-1RA and Insulin Interactions in The Regulation of Lipid Metabolism in NAFLD

As highlighted in the introduction, this review aims to elucidate the role of GLP1 in the direct modulation of molecular pathways of lipid metabolism, beyond its insulinotropic effects in the pancreas. However, a discussion about lipid metabolism cannot exclude the GLP1 and insulin relationship. The effects of insulin on lipid metabolism in the liver have been well established. In summary, in vivo studies of both murine models and in humans suggest that hepatic insulin signaling is needed for hepatic lipid synthesis, as well as for promoting and progression of fatty liver disease during insulin resistance [82]. Besides lipotoxicity and inflammation, insulin resistance is a part of a vicious circle that promotes the development of NAFLD. Each of these processes affect and promote the development and progression of the other [83]. The role of GLP-1 in hepatic lipid metabolism, including crosstalk between GLP-1R and the insulin receptor, has been frequently reported. Furthermore, there is a possible association of GLP-1R with IR as an alternative mechanism in the regulation of their signaling [84]. This may complicate the discussion of the presence of GLP1-Rs on hepatocytes and potentially suggest an interaction of GLP1 and the insulin receptor. However, the evidence for a direct GLP1-R and GLP-1RA interaction in hepatocytes cannot be neglected [21,22,56]. It has been established that GLP-1 improves insulin sensitivity in peripheral tissues [85], while a recent study provided compelling direct and indirect evidence that GLP-1RAs improves insulin resistance and increase the insulin sensitivity of hepatocytes, as well as demonstrating certain advantages of GLP-1RAs over SGLT-2 inhibitors regarding modulating insulin resistance in NAFLD patients [86]. GLP-1 receptor signaling in hepatocytes revealed that exenatide alleviates hepatic steatosis by regulating hepatic insulin clearance through induction in mice, highlighting the fact that increased insulin clearance has been linked to the improvement of NAFLD [87]. 

**Figure 1:**
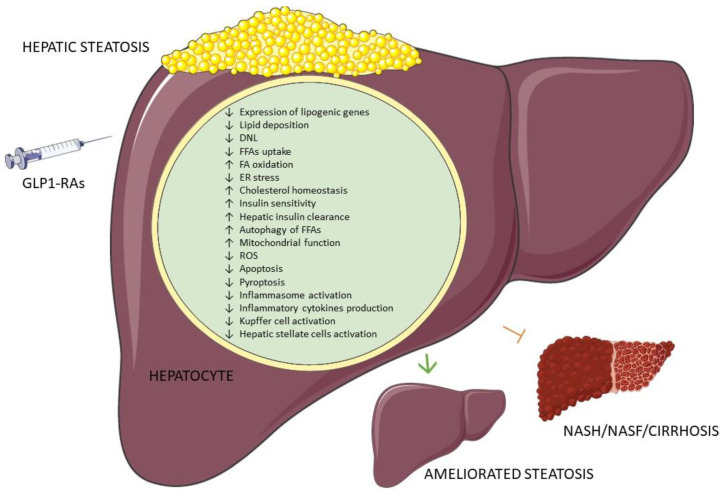
GLP-1RA effects in NAFLD cell culture models. ROS: reactive oxygen species; DNL: de novo lipogenesis; FFAs: free fatty acids; FA: fatty acid; ER: endoplasmic reticulum; GLP-1RA: glucagon-like peptide 1 receptor agonists; NASH: non-alcoholic steatohepatitis; NASF: non-alcoholic steatofibrosis. Green arrow: Processes result in ameliorated steatosis; Red arrow: Processes result in inhibition of NAFLD progression to NASH/NASF/cirrhosis. Figure created with Servier Medical Art, https://smart.servier.com/.

These studies provide evidence for yet another mechanism of the NAFLD-ameliorating GLP1-RA pharmacologic properties, contributing to a plethora of other evidence supporting the role of GLP-1RAs in the treatment of NAFLD. The mechanisms are illustrated in Figure 1.

## 3. Current Status of GLP-1RAs in The Treatment of Progressive NAFLD

As mentioned previously, GLP-1RAs have been approved globally for the treatment of obesity and T2DM, but not for patients with NAFLD, despite resulting in the improvement of NAFLD in patients with these diseases [88,89]. 

A systematic review and meta-analysis of randomized controlled trials regarding the potential of liraglutide in the treatment of NAFLD published in 2021 concluded that despite its promising potential, evidence does not support the administration of liraglutide to patients with NAFLD at the time [35]. A randomized, placebo-controlled phase 2 trial of semaglutide treatment in patients with NASH-related cirrhosis is the most recent study regarding the GLP-1RA treatment of NAFLD. The study concluded that semaglutide 2.4 mg once weekly in patients with NASH-related cirrhosis did not significantly improve fibrosis or NASH versus a placebo. Semaglutide did, however, improve cardiometabolic risk parameters (weight loss, glycemic, and lipids homeostasis) [90]. However, liraglutide and semaglutide have previously been explored in patients with NASH, mainly without cirrhosis. In summary, results showed NASH resolution [91,92] and no worsening of fibrosis [92] versus the placebo group, contrary to the novel study on patients with NASH-induced cirrhosis. 

These novel results, perhaps disappointing, actually provide evidence of the importance of early intervention and the need to provide pharmacologic options, such as GLP1-RAs for the treatment of NAFLD aiming to prevent its progression to NASH, NASF, and cirrhosis. Moreover, a recent case report presented results of liraglutide treatment in obese, pre-diabetic patients not compliant with lifestyle modifications with NASH-induced cirrhosis. Liraglutide demonstrated the reversal of disease progression despite no significant changes in weight, providing further evidence of the importance of pharmacotherapeutic interventions in patients with NASH, as well as suggesting the direct effect of liraglutides on hepatic tissue, independent of pleiotropic effects due to improvements in body mass index [93]. The most recent clinical trial of semaglutide in NAFLD is currently recruiting participants [94]; however, it has shown the efforts of the scientific community in finding pharmacotherapeutic options intended for NAFLD and the recognition of its necessity. 

## 4. Conclusions

Firstly, in this article we provide the most recently obtained knowledge for elucidating the role of GLP1-RAs in the modulation of hepatic lipid metabolism and treatment of NAFLD. While there are similar review articles examining this subject, we opted out of evidence extrapolated from in vitro studies for several particular reasons, predominantly to exclude the pleiotropic effects of GLP-1RAs, to which their beneficial effects are commonly attributed, aiming to distinguish between their indirect and direct effects on hepatocyte hepatic lipid metabolism modulation and provide insight into the “controversial issue” of the presence of GLP-1Rs on hepatocytes. Current evidence shows the ability of GLP-1RAs to directly alleviate hepatic steatosis, modulate lipid metabolism pathways, such as lipogenesis, FA oxidation, and cholesterol secretion, regulate oxidative stress, and reduce lipotoxicity, as well as inflammation. Figure 1 summarizes the cellular and molecular mechanisms underlying these effects, implying a direct role for GLP1-RAs in hepatic tissue, highlighting their potential in patients with NAFLD. These in vitro studies also identified novel potential targets for NAFLD treatment. Aiming to provide a comprehensive review of currently used cell culture models for GLP1RAs in NAFLD treatment research, we have provided Table 1.

Secondly, the most recent clinical trials suggest that early intervention and pharmacological options, such as GLP1-RAs, are vital for limiting NAFLD progression to severe NASH, NASF, and cirrhosis, in addition to off-label use in case reports indicating their success even in progressive NASH with developed cirrhosis.

Interestingly, there is a relative scarcity of in vivo and in vitro studies on semaglutide, the most recently approved GLP-1RA for the treatment of obesity by the FDA, as well as a lack of rapidly evolving 3D cell culture model studies on the role of GLP-1RAs in NAFLD, which mimic in vivo states to a greater extent in comparison to 2D cell culture models, showing the need for further research in this area.

To conclude, in addition to lifestyle changes, such as physical activity and dietary modifications as important adjuncts to pharmacotherapy in managing NAFLD, GLP1-RAs demonstrated the highly efficient modulation and alleviation of hepatic steatosis and aberrant signaling pathways involved in NAFLD pathophysiology and progression in vitro. In addition to the clinical trials, this evidence could be a significant step in acknowledging GLP-1RAs as one of the potential pharmacotherapeutic options for the treatment of NAFLD and its progressive states, which has not been approved and implemented in clinical practice for this disease.

## Figures and Tables

**Table 1:** In vitro experimental cell culture models of NAFLD/NASH/NASF for GLP1-RA treatment research.

Cell Line	Compounds Used to Induce Cell Culture Model of Hepatic Steatosis *	GLP1-RA Compound	Reference
Primary hepatocytes Hep-G2 HuH7	Palmitic acid Oleic acid	GLP-1 Exendin-4	[21]
Huh7	Oleic acid Amiodarone	Liraglutide	[26]
HepG2	Oleic acid	Exendin-4	[49]
HepG2	Oleic acid	AWRK6 (synthetic peptide)	[56]
HepG2	Palmitic acid	Liraglutide	[57,79]
L02	Palmitic acid	Exenatide	[62]
HepG2	High glucose	Liraglutide	[66]
Hepg2	Palmitic acid	Liraglutide	[69]
HepG2	Palmitic acid Oleic acid	Liraglutide	[70]
HepG2	Oleic acid LPS	Exenatide	[75]
Primary mice Kupffer cells (KCs)	Palmitic acid	Liraglutide	[76,77]
AML12 + JS-1	Palmitic acid (AML12) H_2_O_2_ (AML12)	Liraglutide	[78]

* insulin resistance/high glucose environment/NASH.

## Data Availability

Not applicable.

## References

[1] Riazi K., Azhari H., Charette J.H., Underwood F.E., King J.A., Afshar E.E., Swain M.G., Congly S.E., Kaplan G.G., Shaheen A.A. (2022). The prevalence and incidence of NAFLD worldwide: A systematic review and meta-analysis. Lancet Gastroenterol. Hepatol..

[2] Teng M.L., Ng C.H., Huang D.Q., Chan K.E., Tan D.J., Lim W.H., Yang J.D., Tan E., Muthiah M.D. (2023). Global incidence and prevalence of nonalcoholic fatty liver disease. Clin. Mol. Hepatol..

[3] Kumar R., Priyadarshi R.N., Anand U. (2020). Non-alcoholic Fatty Liver Disease: Growing Burden, Adverse Outcomes and Associations. J. Clin. Transl. Hepatol..

[4] Buzzetti E., Pinzani M., Tsochatzis E.A. (2016). The multiple-hit pathogenesis of non-alcoholic fatty liver disease (NAFLD). Metabolism.

[5] Holst J.J. (2007). The physiology of glucagon-like peptide 1. Physiol. Rev..

[6] Crunkhorn S. (2021). Illuminating the incretin effect. Nat. Rev. Endocrinol..

[7] Nauck M.A., Meier J.J. (2016). The incretin effect in healthy individuals and those with type 2 diabetes: Physiology, pathophysiology, and response to therapeutic interventions. Lancet Diabetes Endocrinol..

[8] Perley M.J., Kipnis D.M. (1967). Plasma insulin responses to oral and intravenous glucose: Studies in normal and diabetic sujbjects. J. Clin. Investig..

[9] Knop F.K., Aaboe K., Vilsbøll T., Vølund A., Holst J.J., Krarup T., Madsbad S. (2012). Impaired incretin effect and fasting hyperglucagonaemia characterizing type 2 diabetic subjects are early signs of dysmetabolism in obesity. Diabetes Obes. Metab..

[10] Hare K.J., Vilsbøll T., Holst J.J., Knop F.K. (2010). Inappropriate glucagon response after oral compared with isoglycemic intravenous glucose administration in patients with type 1 diabetes. Am. J. Physiol. Endocrinol. Metab..

[11] Dupre J. (2005). Glycaemic effects of incretins in Type 1 diabetes mellitus: A concise review, with emphasis on studies in humans. Regul. Pept..

[12] Michałowska J., Miller-Kasprzak E., Bogdański P. (2021). Incretin Hormones in Obesity and Related Cardiometabolic Disorders: The Clinical Perspective. Nutrients.

[13] Bagger J.I., Knop F.K., Lund A., Vestergaard H., Holst J.J., Vilsbøll T. (2011). Impaired regulation of the incretin effect in patients with type 2 diabetes. J. Clin. Endocrinol. Metab..

[14] Junker A.E. (2017). The role of incretin hormones and glucagon in patients with liver disease. Dan. Med. J..

[15] Kuhre R.E., Deacon C.F., Holst J.J., Petersen N. (2021). What Is an L-Cell and How Do We Study the Secretory Mechanisms of the L-Cell?. Front. Endocrinol..

[16] Holst J.J. (2019). The incretin system in healthy humans: The role of GIP and GLP-1. Metabolism.

[17] Nauck M.A., Quast D.R., Wefers J., Pfeiffer A.F.H. (2021). The evolving story of incretins (GIP and GLP-1) in metabolic and cardiovascular disease: A pathophysiological update. Diabetes Obes. Metab..

[18] Ding X., Saxena N.K., Lin S., Gupta N.A., Anania F.A. (2006). Exendin-4, a glucagon-like protein-1 (GLP-1) receptor agonist, reverses hepatic steatosis in ob/ob mice. Hepatology.

[19] Panjwani N., Mulvihill E.E., Longuet C., Yusta B., Campbell J.E., Brown T.J., Streutker C., Holland D., Cao X., Baggio L.L. (2013). GLP-1 receptor activation indirectly reduces hepatic lipid accumulation but does not attenuate development of atherosclerosis in diabetic male ApoE^−/−^ mice. Endocrinology.

[20] Lyu J., Imachi H., Fukunaga K., Sato S., Kobayashi T., Dong T., Saheki T., Matsumoto M., Iwama H., Zhang H. (2020). Role of ATP-binding cassette transporter A1 in suppressing lipid accumulation by glucagon-like peptide-1 agonist in hepatocytes. Mol. Metab..

[21] Gupta N.A., Mells J., Dunham R.M., Grakoui A., Handy J., Saxena N.K., Anania F.A. (2010). Glucagon-like peptide-1 receptor is present on human hepatocytes and has a direct role in decreasing hepatic steatosis in vitro by modulating elements of the insulin signaling pathway. Hepatology.

[22] Yokomori H., Ando W. (2020). Spatial expression of glucagon-like peptide 1 receptor and caveolin-1 in hepatocytes with macrovesicular steatosis in non-alcoholic steatohepatitis. BMJ Open Gastroenterol..

[23] Vendrell J., El Bekay R., Peral B., García-Fuentes E., Megia A., Macias-Gonzalez M., Fernández Real J., Jimenez-Gomez Y., Escoté X., Pachón G. (2011). Study of the potential association of adipose tissue GLP-1 receptor with obesity and insulin resistance. Endocrinology.

[24] Thorens B., Porret A., Bühler L., Deng S.P., Morel P., Widmann C. (1993). Cloning and functional expression of the human islet GLP-1 receptor. Demonstration that exendin-4 is an agonist and exendin-(9-39) an antagonist of the receptor. Diabetes.

[25] Mells J.E., Anania F.A. (2013). The role of gastrointestinal hormones in hepatic lipid metabolism. Semin. Liver Dis..

[26] Omanovic Kolaric T., Kizivat T., Mihaljevic V., Zjalic M., Bilic-Curcic I., Kuna L., Smolic R., Vcev A., Wu G.Y., Smolic M. (2022). Liraglutide Exerts Protective Effects by Downregulation of PPARγ, ACSL1 and SREBP-1c in Huh7 Cell Culture Models of Non-Alcoholic Steatosis and Drug-Induced Steatosis. Curr. Issues Mol. Biol..

[27] Trujillo J.M., Nuffer W., Smith B.A. (2021). GLP-1 receptor agonists: An updated review of head-to-head clinical studies. Ther. Adv. Endocrinol. Metab..

[28] Pechenov S., Revell J., Will S., Naylor J., Tyagi P., Patel C., Liang L., Tseng L., Huang Y., Rosenbaum A.I. (2021). Development of an orally delivered GLP-1 receptor agonist through peptide engineering and drug delivery to treat chronic disease. Sci. Rep..

[29] Knudsen L.B., Lau J. (2019). The Discovery and Development of Liraglutide and Semaglutide. Front. Endocrinol..

[30] Idrees Z., Cancarevic I., Huang L. (2022). FDA-Approved Pharmacotherapy for Weight Loss Over the Last Decade. Cureus.

[31] Ghosal S., Datta D., Sinha B. (2021). A meta-analysis of the effects of glucagon-like-peptide 1 receptor agonist (GLP1-RA) in nonalcoholic fatty liver disease (NAFLD) with type 2 diabetes (T2D). Sci. Rep..

[32] Wong C., Lee M.H., Yaow C.Y.L., Chin Y.H., Goh X.L., Ng C.H., Lim A.Y.L., Muthiah M.D., Khoo C.M. (2021). Glucagon-Like Peptide-1 Receptor Agonists for Non-Alcoholic Fatty Liver Disease in Type 2 Diabetes: A Meta-Analysis. Front. Endocrinol..

[33] Zhang F., Chen Z., Wu D., Tian L., Chen Q., Ye Y., Chen W., Wu X., Wu P., Yuan W. (2021). Recombinant human GLP-1 beinaglutide regulates lipid metabolism of adipose tissues in diet-induced obese mice. iScience.

[34] Drucker D.J. (2018). Mechanisms of Action and Therapeutic Application of Glucagon-like Peptide-1. Cell Metab..

[35] Kalogirou M.S., Patoulias D., Haidich A.B., Akriviadis E., Sinakos E. (2021). Liraglutide in patients with non-alcoholic fatty liver disease: A systematic review and meta-analysis of randomized controlled trials. Clin. Res. Hepatol. Gastroenterol..

[36] Xie Z., Yang S., Deng W., Li J., Chen J. (2022). Efficacy and Safety of Liraglutide and Semaglutide on Weight Loss in People with Obesity or Overweight: A Systematic Review. Clin. Epidemiol..

[37] Verma S., Bhatt D.L., Bain S.C., Buse J.B., Mann J.F.E., Marso S.P., Nauck M.A., Poulter N.R., Pratley R.E., Zinman B. (2018). Effect of Liraglutide on Cardiovascular Events in Patients with Type 2 Diabetes Mellitus and Polyvascular Disease: Results of the LEADER Trial. Circulation.

[38] Singh G., Krauthamer M., Bjalme-Evans M. (2022). Wegovy (semaglutide): A new weight loss drug for chronic weight management. J. Investig. Med..

[39] Girdhar K., Dehury B., Kumar Singh M., Daniel V.P., Choubey A., Dogra S., Kumar S., Mondal P. (2019). Novel insights into the dynamics behavior of glucagon-like peptide-1 receptor with its small molecule agonists. J. Biomol. Struct. Dyn..

[40] Andersen A., Lund A., Knop F.K., Vilsbøll T. (2018). Glucagon-like peptide 1 in health and disease. Nat. Rev. Endocrinol..

[41] Jianping W., Xuelian Z., Anjiang W., Haiying X. (2021). Efficacy and Safety of Glucagon-like Peptide-1 Receptor Agonists in the Treatment of Metabolic Associated Fatty Liver Disease: A Systematic Review and Meta-analysis. J. Clin. Gastroenterol..

[42] Barritt A.S., Marshman E., Noureddin M. (2022). Review article: Role of glucagon-like peptide-1 receptor agonists in non-alcoholic steatohepatitis, obesity and diabetes-what hepatologists need to know. Aliment. Pharmacol. Ther..

[43] Yaribeygi H., Maleki M., Butler A.E., Jamialahmadi T., Sahebkar A. (2021). The Impact of Incretin-Based Medications on Lipid Metabolism. J. Diabetes Res..

[44] Farr S., Taher J., Adeli K. (2014). Glucagon-like peptide-1 as a key regulator of lipid and lipoprotein metabolism in fasting and postprandial states. Cardiovasc. Hematol. Disord. Drug. Targets.

[45] Villanueva-Peñacarrillo M.L., Márquez L., González N., Díaz-Miguel M., Valverde I. (2001). Effect of GLP-1 on lipid metabolism in human adipocytes. Horm. Metab. Res..

[46] Sofogianni A., Filippidis A., Chrysavgis L., Tziomalos K., Cholongitas E. (2020). Glucagon-like peptide-1 receptor agonists in non-alcoholic fatty liver disease: An update. World J. Hepatol..

[47] Armstrong M., Hull D., Guo K., Barton D., Yu J., Tomlinson J., Newsome P. (2014). Effect of liraglutide on adipose insulin resistance and hepatic de-novo lipogenesis in non-alcoholic steatohepatitis: Substudy of a phase 2, randomised placebo-controlled trial. Lancet.

[48] Armstrong M.J., Hull D., Guo K., Barton D., Hazlehurst J.M., Gathercole L.L., Nasiri M., Yu J., Gough S.C., Newsome P.N. (2016). Glucagon-like peptide 1 decreases lipotoxicity in non-alcoholic steatohepatitis. J. Hepatol..

[49] Khalifa O., Al-Akl N.S., Errafii K., Arredouani A. (2022). Exendin-4 alleviates steatosis in an in vitro cell model by lowering FABP1 and FOXA1 expression via the Wnt/-catenin signaling pathway. Sci. Rep..

[50] Wang Y., Viscarra J., Kim S.J., Sul H.S. (2015). Transcriptional regulation of hepatic lipogenesis. Nat. Rev. Mol. Cell Biol..

[51] Sookoian S., Pirola C.J., Valenti L., Davidson N.O. (2020). Genetic Pathways in Nonalcoholic Fatty Liver Disease: Insights From Systems Biology. Hepatology.

[52] Moya M., Benet M., Guzmán C., Tolosa L., García-Monzón C., Pareja E., Castell J.V., Jover R. (2012). Foxa1 reduces lipid accumulation in human hepatocytes and is down-regulated in nonalcoholic fatty liver. PLoS ONE.

[53] Guzmán C., Benet M., Pisonero-Vaquero S., Moya M., García-Mediavilla M.V., Martínez-Chantar M.L., González-Gallego J., Castell J.V., Sánchez-Campos S., Jover R. (2013). The human liver fatty acid binding protein (FABP1) gene is activated by FOXA1 and PPARα; and repressed by C/EBPα: Implications in FABP1 down-regulation in nonalcoholic fatty liver disease. Biochim. Biophys. Acta.

[54] Fang C., Pan J., Qu N., Lei Y., Han J., Zhang J., Han D. (2022). The AMPK pathway in fatty liver disease. Front. Physiol..

[55] Miao R., Fang X., Wei J., Wu H., Wang X., Tian J. (2022). Akt: A Potential Drug Target for Metabolic Syndrome. Front. Physiol..

[56] Jin L., Sun Y., Li Y., Zhang H., Yu W., Xin Y., Alsareii S.A., Wang Q., Zhang D. (2021). A synthetic peptide AWRK6 ameliorates metabolic associated fatty liver disease: Involvement of lipid and glucose homeostasis. Peptides.

[57] Yu P., Xu X., Zhang J., Xia X., Xu F., Weng J., Lai X., Shen Y. (2019). Liraglutide Attenuates Nonalcoholic Fatty Liver Disease through Adjusting Lipid Metabolism via SHP1/AMPK Signaling Pathway. Int. J. Endocrinol..

[58] Sharma Y., Ahmad A., Yavvari P.S., Kumar Muwal S., Bajaj A., Khan F. (2019). Targeted SHP-1 Silencing Modulates the Macrophage Phenotype, Leading to Metabolic Improvement in Dietary Obese Mice. Mol. Ther. Nucleic Acids.

[59] Dubois M.J., Bergeron S., Kim H.J., Dombrowski L., Perreault M., Fournès B., Faure R., Olivier M., Beauchemin N., Shulman G.I. (2006). The SHP-1 protein tyrosine phosphatase negatively modulates glucose homeostasis. Nat. Med..

[60] Lin L., Jian J., Song C.Y., Chen F., Ding K., Xie W.F., Hu P.F. (2020). SHP-1 ameliorates nonalcoholic steatohepatitis by inhibiting proinflammatory cytokine production. FEBS Lett..

[61] Xu E., Forest M.P., Schwab M., Avramoglu R.K., St-Amand E., Caron A.Z., Bellmann K., Shum M., Voisin G., Paquet M. (2014). Hepatocyte-specific Ptpn6 deletion promotes hepatic lipid accretion, but reduces NAFLD in diet-induced obesity: Potential role of PPARγ. Hepatology.

[62] Li S., Wang X., Zhang J., Li J., Liu X., Ma Y., Han C., Zhang L., Zheng L. (2018). Exenatide ameliorates hepatic steatosis and attenuates fat mass and FTO gene expression through PI3K signaling pathway in nonalcoholic fatty liver disease. Braz. J. Med. Biol. Res..

[63] Favari E., Chroni A., Tietge U.J., Zanotti I., Escolà-Gil J.C., Bernini F. (2015). Cholesterol efflux and reverse cholesterol transport. Handb. Exp. Pharmacol..

[64] Yousefi B., Darabi M., Baradaran B., Khaniani M.S., Rahbani M., Fayezi S., Mehdizadeh A., Saliani N., Shaaker M. (2012). Inhibition of MEK/ERK1/2 Signaling Affects the Fatty Acid Composition of HepG2 Human Hepatic Cell Line. Bioimpacts.

[65] Kassouf T., Sumara G. (2020). Impact of Conventional and Atypical MAPKs on the Development of Metabolic Diseases. Biomolecules.

[66] Wu Y.R., Shi X.Y., Ma C.Y., Zhang Y., Xu R.X., Li J.J. (2019). Liraglutide improves lipid metabolism by enhancing cholesterol efflux associated with ABCA1 and ERK1/2 pathway. Cardiovasc. Diabetol..

[67] Schulze R.J., Krueger E.W., Weller S.G., Johnson K.M., Casey C.A., Schott M.B., McNiven M.A. (2020). Direct lysosome-based autophagy of lipid droplets in hepatocytes. Proc. Natl. Acad. Sci. USA.

[68] Fang Y., Ji L., Zhu C., Xiao Y., Zhang J., Lu J., Yin J., Wei L. (2020). Liraglutide Alleviates Hepatic Steatosis by Activating the TFEB-Regulated Autophagy-Lysosomal Pathway. Front. Cell Dev. Biol..

[69] He Y., Ao N., Yang J., Wang X., Jin S., Du J. (2020). The preventive effect of liraglutide on the lipotoxic liver injury via increasing autophagy. Ann. Hepatol..

[70] Zhang Q., Liu Q., Niu C.Y. (2021). Liraglutide alleviates lipotoxic liver cell damage and promotes autophagy to improve non-alcoholic fatty liver. Zhonghua Gan Zang Bing Za Zhi.

[71] Arroyave-Ospina J.C., Wu Z., Geng Y., Moshage H. (2021). Role of Oxidative Stress in the Pathogenesis of Non-Alcoholic Fatty Liver Disease: Implications for Prevention and Therapy. Antioxidants.

[72] Cusi K. (2012). Role of obesity and lipotoxicity in the development of nonalcoholic steatohepatitis: Pathophysiology and clinical implications. Gastroenterology.

[73] Huang C., Yuan L., Cao S. (2015). Endogenous GLP-1 as a key self-defense molecule against lipotoxicity in pancreatic islets. Int. J. Mol. Med..

[74] Somm E., Montandon S.A., Loizides-Mangold U., Gaïa N., Lazarevic V., De Vito C., Perroud E., Bochaton-Piallat M.-L., Dibner C., Schrenzel J. (2021). The GLP-1R agonist liraglutide limits hepatic lipotoxicity and inflammatory response in mice fed a methionine-choline deficient diet. Transl. Res..

[75] Liu Y., Wang D.W., Wang D., Duan B.H., Kuang H.Y. (2021). Exenatide Attenuates Non-Alcoholic Steatohepatitis by Inhibiting the Pyroptosis Signaling Pathway. Front. Endocrinol..

[76] Zhu W., Feng P.P., He K., Li S.W., Gong J.P. (2018). Liraglutide protects non-alcoholic fatty liver disease via inhibiting NLRP3 inflammasome activation in a mouse model induced by high-fat diet. Biochem. Biophys. Res. Commun..

[77] Li Z., Feng P.P., Zhao Z.B., Zhu W., Gong J.P., Du H.M. (2019). Liraglutide protects against inflammatory stress in non-alcoholic fatty liver by modulating Kupffer cells M2 polarization via cAMP-PKA-STAT3 signaling pathway. Biochem. Biophys. Res. Commun..

[78] Ji J., Feng M., Huang Y., Niu X. (2022). Liraglutide inhibits receptor for advanced glycation end products (RAGE)/reduced form of nicotinamide-adenine dinucleotide phosphate (NAPDH) signaling to ameliorate non-alcoholic fatty liver disease (NAFLD) in vivo and vitro. Bioengineered.

[79] Ao N., Ma Z., Yang J., Jin S., Zhang K., Luo E., Du J. (2020). Liraglutide ameliorates lipotoxicity-induced inflammation through the mTORC1 signalling pathway. Peptides.

[80] Feng J., Qiu S., Zhou S., Tan Y., Bai Y., Cao H., Guo J., Su Z. (2022). mTOR: A Potential New Target in Nonalcoholic Fatty Liver Disease. Int. J. Mol. Sci..

[81] Chen H. (2020). Nutrient mTORC1 signaling contributes to hepatic lipid metabolism in the pathogenesis of non-alcoholic fatty liver disease. Liver Res..

[82] Titchenell P.M., Lazar M.A., Birnbaum M.J. (2017). Unraveling the Regulation of Hepatic Metabolism by Insulin. Trends Endocrinol. Metab..

[83] Chen Z., Yu R., Xiong Y., Du F., Zhu S. (2017). A vicious circle between insulin resistance and inflammation in nonalcoholic fatty liver disease. Lipids Health Dis..

[84] Wang Y., Song X., Wang N. (2022). Specific interaction of insulin receptor and GLP-1 receptor mediates crosstalk between their signaling. Biochem. Biophys. Res. Commun..

[85] Jiang Y., Wang Z., Ma B., Fan L., Yi N., Lu B., Wang Q., Liu R. (2018). GLP-1 Improves Adipocyte Insulin Sensitivity Following Induction of Endoplasmic Reticulum Stress. Front. Pharmacol..

[86] Yan H., Huang C., Shen X., Li J., Zhou S., Li W. (2022). GLP-1 RAs and SGLT-2 Inhibitors for Insulin Resistance in Nonalcoholic Fatty Liver Disease: Systematic Review and Network Meta-Analysis. Front. Endocrinol..

[87] Czech T.Y., Wang Q., Seki E. (2018). A new mechanism of action of glucagon-like peptide-1 agonist in hepatic steatosis: Promotion of hepatic insulin clearance through induction of carcinoembryonic antigen-related cell adhesion molecule 1. Hepatol. Commun..

[88] Zhao Y., Zhao W., Bu H., Toshiyoshi M. (2023). Liraglutide on type 2 diabetes mellitus with nonalcoholic fatty liver disease: A systematic review and meta-analysis of 16 RCTs. Medicine.

[89] Song T., Jia Y., Li Z., Wang F., Ren L., Chen S. (2021). Effects of Liraglutide on Nonalcoholic Fatty Liver Disease in Patients with Type 2 Diabetes Mellitus: A Systematic Review and Meta-Analysis. Diabetes Ther..

[90] Loomba R., Abdelmalek M.F., Armstrong M.J., Jara M., Kjær M.S., Krarup N., Lawitz E., Ratziu V., Sanyal A.J., Schattenberg J.M. (2023). Semaglutide 2·4 mg once weekly in patients with non-alcoholic steatohepatitis-related cirrhosis: A randomised, placebo-controlled phase 2 trial. Lancet Gastroenterol. Hepatol..

[91] Armstrong M.J., Gaunt P., Aithal G.P., Barton D., Hull D., Parker R., Hazlehurst J.M., Guo K., Abouda G., Aldersley M.A. (2016). Liraglutide safety and efficacy in patients with non-alcoholic steatohepatitis (LEAN): A multicentre, double-blind, randomised, placebo-controlled phase 2 study. Lancet.

[92] Newsome P.N., Buchholtz K., Cusi K., Linder M., Okanoue T., Ratziu V., Sanyal A.J., Sejling A.S., Harrison S.A. (2021). A Placebo-Controlled Trial of Subcutaneous Semaglutide in Nonalcoholic Steatohepatitis. N. Engl. J. Med..

[93] Yuen C., Yu T., French S., Marcus E.A., Yeh J., Chiu H. (2023). Treatment of an Adolescent Female With Nonalcoholic Steatohepatitis–Related Cirrhosis with Liraglutide. JPGN Rep..

[94] ClinicalTrials.gov (2023). NCT05813249. NCT05813249.

